# Pronostic obstétrical des femmes atteintes de vaginisme primaire

**DOI:** 10.11604/pamj.2019.32.160.16083

**Published:** 2019-04-08

**Authors:** Elise Tourrilhes, Marie Veluire, David Hervé, Erdogan Nohuz

**Affiliations:** 1 Service de Gynécologie Obstétrique, Centre Hospitalier de Fougères, Fougères, France; 2 Service de Gynécologie Obstétrique, Hôpital Privé d’Athis-Mons, Caron, 91200 Athis Mons, France; 3 Université Clermont-Auvergne, Place Henri Dunant, 63000 Clermont-Ferrand, France; 4 Service de Gynécologie Obstétrique, Centre Hospitalier de Thiers, Route du Fau, 63300 Thiers, France

**Keywords:** Vaginisme, dysfonction sexuelle, dyspareunie, phobie sexuelle, rapport sexuel, accouchement, césarienne, Vaginismus, sexual dysfunction, dyspareunia, sexual phobia, sexual intercourse, childbirth, cesarean section

## Abstract

**Introduction:**

Dysfonction importante, le vaginisme est un problème tant individuel que du couple qui peut altérer la relation sexuelle. Il peut influencer le pronostic obstétrical. Le but de cette étude était de déterminer si les caractéristiques cliniques du vaginisme ont une incidence sur le processus de l’accouchement.

**Méthodes:**

Etude rétrospective multicentrique incluant des patientes affectées par un vaginisme primaire, ayant donné naissance à terme à un premier enfant vivant, entre 2005 et 2015.

**Résultats:**

Sur les 19 patientes inclues dans l’étude, nous avons constaté 9 grossesses prolongées, 14 mises en travail spontanées (dont 8 à terme atteint), 3 césariennes en dehors du travail et 2 déclenchements artificiels. Parmi les 16 femmes ayant eu un travail, nous avons dénombré 4 césariennes, 5 accouchements par voie basse avec forceps et 7 par voie basse spontanée. Parmi les 12 accouchements par voie basse, 9 épisiotomies ont été pratiquées, 7 femmes ont présenté des déchirures périnéales spontanées seules ou associées à une épisiotomie, aucune lésion périnéale de type 3 ou 4, ni de périnées intacts n’ont été recensés. Le poids moyen des nouveau-nés était de 3380g±332 (2870g-3970g, 47^e^ percentile).

**Conclusion:**

La proportion d’accouchements dystociques et la morbidité périnéale étaient notablement élevées, ce qui parait comparable avec la plupart des données de la littérature. Il est possible que les composantes psychologiques et comportementales du vaginisme, (mécanisme de peur-évitement, anxiété) aient favorisé les grossesses prolongées, les césariennes, les dystocies mécaniques et les lésions périnéales. Des études complémentaires sont nécessaires afin de mieux cerner le vaginisme et ses implications obstétricales.

## Abstract (en)

**Introduction:**

Vaginismus is a severe dysfunction and a problem which can interfere with woman’s and couple’s sex life. It may influence the obstetric outcome. This study aims to determine if the clinical features of vaginismus can impact childbirth experience.

**Methods:**

We conducted a retrospective multicenter study involving patients affected by primary vaginismus, having given birth to their first child (who had reached term), between 2005 and 2015.

**Results:**

Out of 19 patients included in the study, 9 had prolonged pregnancies, 14 had spontaneous labor (including 8 at term), 3 had cesarean section before going into labor and 2 had labor induction. Among the 16 women who experienced labor, 4 had cesarean section, 5 had vaginal delivery with the help of forceps and 7 had spontaneous vaginal delivery. Among the 12 women who had vaginal delivery, 9 underwent episiotomy, 7 had spontaneous perineal tear alone or in combination with episiotomy. No 3^rd^ and 4^th^ degree perineal injury or intact perineum were found. The average birth weight for babies was 3380 g ± 332 (2870 g-3970g, 47^th^ percentile).

**Conclusion:**

The rates of labour dystocia and perineal morbidity were significantly high. These data were comparable to most of the data in the literature. It is likely that the psychological and behavioral aspects of vaginismus (fear-avoidance and anxiety-inducing mechanism) have favoured prolonged pregnancies, cesarean sections, mechanical dystocias and perineal injuries. Additional studies are necessary to better identify vaginismus and its obstetrical implications.

## Introduction

Le vaginisme se traduit par la difficulté persistante ou récurrente, pour une femme, de permettre l’entrée de son vagin à un pénis, un doigt et/ou à d’autres objets, en dépit du désir exprimé d’y parvenir. Le tonus accru et incontrôlé des muscles périnéaux a été incriminé dans cette impossibilité de pénétration vaginale bien que des études électromyographiques n’en aient pas totalement élucidé la physiopathologie. Les caractéristiques psychologiques et physiques des femmes atteintes de vaginisme rendent difficile l’accès à une sexualité satisfaisante, mais interrogent également sur leur rôle au moment de l’accouchement [1-5]. Ces femmes sont sujettes à de fréquentes erreurs dans la perception de leur schéma corporel proprioceptif ainsi que de leurs cognitions qui se traduisent par une anxiété majorée et une peur dramatisée des douleurs. Les troubles dans la gestion émotionnelle de leurs sentiments peuvent alors parfois conduire à l’évitement des situations d’intimité [2, 3, 5-13]. Ayant établi ce constat, nous avons souhaité déterminer si le vaginisme pouvait avoir un impact sur le pronostic obstétrical, l’accouchement impliquant en lui-même « le corps sexué », diverses émotions et une grande interaction avec les professionnels de santé.

## Méthodes

Il s’agit d’une étude rétrospective sur l’association entre grossesse et vaginisme primaire. Les patientes incluses dans cette étude étaient des femmes qui, au moment de leur désir d’enfant, n’avaient jamais pu avoir de pénétration vaginale par un pénis (sans obstacle anatomique) et qui ont donné naissance à un premier enfant vivant à terme. Ont été inclues des patientes ayant accouché entre le 1^er^ janvier 2004 et le 30 avril 2015. La période de recueil des données s’échelonnait du 1^er^ septembre 2014 au 30 avril 2015. Nous avons établi un questionnaire de recueil des données obstétricales rempli rétrospectivement par les sages-femmes et les gynécologues-obstétriciens ayant pris en charge ces patientes. Celui-ci comportait des questions relatives aux données sociodémographiques (âge, profession, niveau d’étude), au type d’établissement, aux données obstétricales et néonatales (parité, méthode de procréation, choix de préparation à la naissance et à la parentalité, terme et mode d’accouchement, durée et mode de mise en travail, méthodes d’analgésies, types de lésions périnéales, motifs d’intervention médicale, poids et sexe du nouveau-né) ainsi qu’aux données sexologiques (durée et modalités d’un éventuel suivi sexologique avant l’accouchement, cotation du tonus musculaire périnéal en début de grossesse et avant l’accouchement). Un score de cotation du tonus musculaire périnéal, inspiré de Pacik [14], était évalué sur 5 niveaux: 1 = spasme des releveurs, disparaissant en rassurant la patiente; 2 = spasme des releveurs, persistant lors des examens gynécologiques; 3 = spasme des releveurs, contraction des fesses lors de toute tentative d’examen; 4 = spasme des releveurs, contraction dorsale en arc, adduction des cuisses, mouvements de défense et rétraction des membres inférieurs; 5 = niveau 4 associé à des manifestations végétatives, refus de tout examen. Les professionnels de santé sollicités exerçaient dans divers départements français. Les croissances pondérales ont été calculées à l’aide des courbes [15], les moyennes, médianes, pourcentages et écart-types à l’aide du logiciel Excel^®^. Bien que la taille de l’échantillon fut modeste (ne permettait pas de faire des statistiques inférentielles), des comparaisons intra-échantillon ont pu être effectuées en recourant au test exact de Fisher bilatéral, avec un risque alpha = 5%.

## Résultats

L’échantillon définitif était constitué de 19 femmes ayant accouché en Bretagne et en Île de France, majoritairement en établissement privé à but lucratif (n = 14). La moyenne d’âge à l’accouchement était de 29,5 ans ± 4,5, (extrêmes de 22 à 40 ans). Le Tableau 1 présente les moyennes des scores de cotation du périnée des patientes de l’échantillon.

**Tableau 1:** Scores de cotation du tonus périnéal de l’échantillon (moyenne ± écart-type)

	Echantillon (n = 19)	Patientes avec accompagnement sexologique (n=13)	Patientes sans accompagnement sexologique (n=6)
Score début de grossesse	3,42 ± 0,90	3,24 ± 0,93	3,83 ± 0,75
Score fin de grossesse	2,00 ± 1,08	1,62 ± 0,87	3,00 ± 0,89
Evolution du score	-1.42 ± 1,21	-1,62 ± 1,19	-0,83 ± 1,17

### Grossesse

Deux couples ont conçu par procréation médicalement assistée (FIV) pour un motif autre que le vaginisme, 4 par éjaculation à la vulve (dont une grossesse non désirée), 7 par éjaculation vestibulaire à l’entrée du vagin, 3 par “auto-insémination maison”, 3 données étaient manquantes. Par ailleurs, 16 patientes ont suivi une préparation à la naissance et à la parentalité. Une patiente avait un diabète gestationnel (équilibré sous régime), une a rompu les membranes plus de 12 heures avant l’accouchement, ces situations ayant conduit à un déclenchement artificiel du travail. Parmi les 14 patientes ayant eu un début de travail spontané, 8 (5^7^,1%) avaient atteint le terme (= 41SA). Deux déclenchements artificiels du travail (10,52%) ont été recensés.

### Travail et accouchement

**Figure 1:**
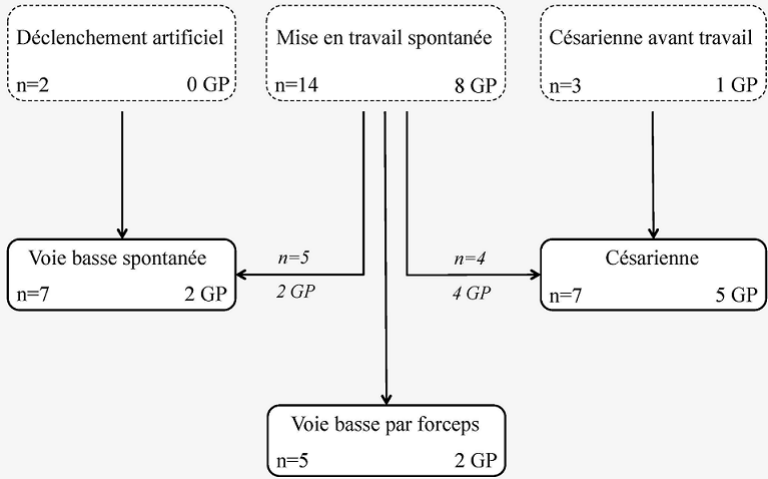
Mise en travail et issue des accouchements des 19 parturientes de l’échantillon, nombre de grossesses prolongées (GP)

La Figure 1 schématise la répartition des 19 parturientes selon leur mode de mise en travail et la répartition des 9 grossesses prolongées. Une analgésie périmédullaire a été réalisée chez 15 parturientes, une patiente n’a bénéficié d’aucune analgésie tandis qu’une anesthésie générale a été pratiquée, en complément de la péridurale afin de procéder à l’extraction fœtale chez une patiente “agitée”. L’on retrouvait 12 accouchements par voie basse et 7 accouchements par césarienne répartis selon: 1) 12 accouchements par voie basse (63,2%) dont 7 accouchements par voie basse spontanée et 5 accouchements par voie basse instrumentale; 2) 7 césariennes (36,8%) dont 4 césariennes programmées avant travail et 3 césariennes en cours de travail.

Les indications des césariennes programmées étaient le vaginisme sans mise en travail dans un contexte de terme atteint (n = 2) et le sauvetage fœtal (n = 1) tandis que celles réalisées en cours de parturition l’étaient du fait d’une dystocie cervicale (n = 2) et d’une altération du rythme cardiaque fœtal (n = 2). Les extractions instrumentales (41,6% des accouchements par voie basse) étaient effectuées pour non progression du mobile fœtal (n = 3), insuffisance de poussées expulsives (n = 1) et agitation maternelle (n = 1). La durée moyenne de la phase active du deuxième stade du travail (poussées expulsives) était de 19,6 minutes (± 8,9 min).

### Périnée

Une lésion périnéale a été retrouvée chez toutes les patientes qu’il s’agisse d’épisiotomie ou de déchirure vulvo-périnéales. Ainsi, une épisiotomie était réalisée chez 9 patientes (75%) pour extraction instrumentale (n = 2), altération du rythme cardiaque fœtal (n = 2), hypertonie périnéale (n = 3). Deux données étaient manquantes (concernant des accouchements par voie basse spontanés). L’on retrouvait une déchirure périnéale chez 7 parturientes (58,3%). Une périnéorraphie a nécessité une anesthésie générale et s’est compliquée d’une hémorragie sévère (1litre). L’état périnéal selon le mode d’accouchement par voie basse est rapporté dans le Tableau 2.

**Tableau 2:** Morbidité périnéale immédiate et nombre de patientes concernées selon le type d’accouchement voie basse

	Spontané (n = 7)	Forceps (n = 5)
Périnée intact	0	0
Lésion vulvaire type éraillure, suturée	1	0
Déchirure périnéale simple (type 1 ou 2)	1	0
Déchirure périnéale complète (type 3 ou 4)	0	0
Episiotomie isolée	2	4
Episiotomie associée à une déchirure périnéale simple	2	1
Déchirure cervicale associée à de multiples déchirures vaginales	1	0

### Nouveau-nés

La moyenne des poids de naissance des nouveau-nés (10 garçons et 9 filles) était de 3380g ± 331,9 (extrêmes 2870g - 3970g), situant le percentile au 47,2^e^ ± 25,6, (extrêmes 8,25-88,76).

### Tests statistiques

Nous avons trouvé une association significative entre l’existence d’un accompagnement sexologique et une absence d’hypertonie périnéale en fin de grossesse (score à 1/5), p = 0,04. Nous n’avons, en revanche, pas trouvé d’association significative entre: 1) la présence d’une hypertonie périnéale en fin de grossesse (score entre 2 et 4/5) et le recours aux forceps, p = 0,29; 2) la grossesse prolongée et l’accouchement non physiologique (forceps ou césarienne), p = 0,35; 3) l’existence d’un accompagnement sexologique et un accouchement physiologique (en excluant les interventions pour sauvetage fœtal), p = 0,37; 4) le fait d’avoir suivi une préparation à la naissance et la réduction du score de tonus périnéal (p = 0,53), ni avec l’accouchement physiologique (p = 0,52); 5) la réduction du score du tonus périnéal et l’accouchement physiologique (p = 0,60); 6) le poids de naissance supérieur à 3600g et l’accouchement non physiologique (forceps ou césarienne) (p = 1).

## Discussion

### Population

Les données sociodémographiques de notre échantillon étaient semblables à celles de l’enquête nationale périnatale de 2016 [16] pour les niveaux d’études et la répartition dans les catégories socioprofessionnelles. Mais un biais était introduit par le fait que les femmes avaient majoritairement accouché en établissement privé à but lucratif, plutôt qu’en structure publique. L’âge moyen des femmes de notre cohorte et de celles d’autres auteurs est semblable: Möller 24 à 29 ans [17] et Drenth 28 ans [18]. En France, l’âge moyen au premier enfant en France était de 28,5 ans et, tous rangs de naissance confondus, de 30,4 ans [19]. Contrairement à ce que les difficultés de procréation pourraient laisser croire, les femmes atteintes de vaginisme ne semblent pas avoir tendance à concevoir leur premier enfant plus tardivement que l’ensemble des femmes. Goldsmith *et al.*rapportent même une moyenne d’âge paraissant significativement moindre (bien que la cohorte comprenne plus de patientes primipares) [20].

### Grossesse, nouveau-nés

Parmi les patientes ayant eu un début de travail spontané, la proportion de celles ayant atteint le terme (= 41SA) était considérable. En comparaison, ce taux se situe aux alentours de 15% dans la littérature [16, 21]. Une grande proportion de grossesses prolongées chez les patientes vaginiques a été constatée par Quiret-Rousselle [22], mais cette donnée n’a pas été mentionnée par les autres auteurs [17, 18, 20]. Ce résultat interroge sur la participation des facteurs cognitifs et émotionnels dans la mise en travail, notamment l’influence de la peur. Malgré tout, peu de déclenchements artificiels du travail ont été recensés dans notre population, contrairement à ce qui a été constaté par Quiret-Rousselle (61,53%) et Goldsmith *et al.* (37,3%).

Les nouveau-nés étaient tous normotrophes, et nous n’avons pas trouvé d’association entre le poids supérieur à 3600g et l’accouchement par césarienne ou forceps (p = 1). Si Goldsmith *et al.* [20] ont rapporté des nouveau-nés plus petits, ils auraient retrouvé aussi significativement plus de naissances induites. Ceci pourrait en partie être dû à une restriction de croissance à moins qu’un biais introduit par des naissances à terme plus avancé ne puisse l’expliquer. Les croissances pondérales de notre échantillon étaient légèrement inférieures au 50^è^percentile, bien que nous ne disposions pas de tous les éléments pour interpréter ce résultat, notamment s’agissant des attitudes addictives comme la consommation de toxiques et du degré d’anxiété des patientes [23].

### Accouchement

Si une grande majorité des patientes de notre échantillon a eu recours à l’analgésie péridurale, celle-ci n’a pas toujours permis de faciliter les examens pelviens. Il faut toutefois rappeler que ce mode d’analgésie périmédullaire n’est pas un facteur de risque de césarienne en cours de travail ni d’extractions instrumentales [24]. Le vaginisme comme motif principal des césariennes avant travail a sans doute contribué à majorer le taux de césarienne dans notre échantillon. L’enquête nationale périnatale retrouvait ce taux à 20,2% pour les femmes à terme et 23,4% pour les primipares quel que soit le terme [16]. Le risque de césarienne apparait significativement supérieur chez les femmes atteintes de vaginisme [4, 17, 20, 22]. Dans la littérature, les césariennes avant travail attribuées au vaginisme s’expliquaient par la forte demande de césarienne de convenance, en raison d’une peur de l’accouchement [17, 20], et par l’impossibilité de pratiquer des déclenchements artificiels [20]. Les auteurs expliquaient les césariennes en cours de travail par les difficultés à examiner les parturientes, sources de retard de diagnostique [17, 20], notamment pour ce qui est des disproportions fœto-pelviennes [20]. Néanmoins, ceci est à interpréter avec circonspection dans la mesure où les poids de naissance apparaissaient significativement inférieurs [20]. Par ailleurs, les césariennes en cours de travail ont été effectuées dans un contexte de grossesse prolongée qui est en soi reconnu comme un facteur de risque de césarienne [21]. La grossesse prolongée expliquerait donc en partie notre taux élevé de césariennes. Toutefois, 2 d’entre elles ont été réalisées pour une dystocie cervicale dans la phase active du premier stade du travail; l’hypothèse de la participation de la peur de l’accouchement dans la survenue de ces césariennes en cours de travail pourrait dès lors être discutée [25].

Le taux d’extractions instrumentales est également apparu élevé dans notre échantillon. Ce constat est également déploré Quiret-Rousselle (46,2%) [22]. Le taux révélé par l’enquête nationale périnatale était de 12,2% pour les parturientes à terme [16]. Möller *et al.* [17] avaient une proportion d’extraction instrumentales non significativement supérieure, dans un échantillon qui, rappelons-le, comportait indifféremment des femmes souffrant de vaginisme et de vulvodynie. En revanche, Goldsmith *et al.* [20] avaient calculé un risque d’extraction instrumentale multiplié par 3,6 en cas de vaginisme, qu’ils expliquaient par l’incapacité pour les patientes de pousser vigoureusement lors du second stade du travail. De fait, dans notre échantillon, les forceps ont été pratiqués sur des fœtus n’excédant pas 3440g, et la non-progression du mobile fœtal pourrait s’expliquer par une insuffisance de poussée, soit en raison d’une altération des perceptions et du contrôle du périnée, soit en raison d’un excès de tonus musculaire du périnée profond, voire en raison d’une peur. Les forceps pratiqués pour non-progression du mobile fœtal et insuffisance d’efforts expulsifs, l’ont été pour des patientes ayant eu un accompagnement sexologique, donc un apprentissage des sensations et du contrôle périnéal. Même si dans la population apparaissait une association significative entre l’accompagnement sexologique et l’absence d’hypertonie périnéale en fin de grossesse, l’on pouvait trouver une association non significative entre la présence d’une hypertonie périnéale en fin de grossesse et le recours au forceps. Effectivement, les scores de fin de grossesse étaient situés entre 1 et 4, et leur évolution n’a pas toujours été dans le sens d’une amélioration (de -3 à +1). Ces femmes n’avaient donc pas toutes une hypertonie périnéale constante. Le point commun des patientes ayant eu un accouchement avec extraction instrumentale par forceps pouvait être un mécanisme de peur-évitement, au cœur de la clinique du vaginisme. L’état de panique manifesté par une des parturientes en est l’apogée même si le vaginisme ne détient certes pas le monopole de ce type de manifestation. La peur de l’expulsion du fœtus pourrait conduire à un évitement de la poussée, et/ou un hyper-tonus des muscles du périnée profond contre lesquels le mobile fœtal viendrait butter.

L’hypertonie musculaire périnéale pourrait aussi expliquer la morbidité périnéale particulière des femmes de notre échantillon puisque toutes les patientes ont présenté une lésion périnéale. Quiret-Rousselle *et al.*constatent aussi un taux de lésions périnéales chez toutes le patientes de leur cohorte [22]. Möller *et al.*confirment une augmentation significative du risque de lésions périnéales dans leur étude [17], bien que Goldsmith *et al.* ne l’évoquent pas [20]. En comparaison, l’enquête nationale périnatale retrouve 34,9% d’épisiotomies chez les primoparturientes et 52% de lésions périnéales spontanées pour l’ensemble des femmes ayant accouché par voie basse [16]. Il est hasardeux de tenter d’expliquer la survenue de déchirures spontanées au cours de l’accouchement d’une femme vaginique, en raison de l’intrication de nombreux paramètres. Le poids de naissance des enfants ne saurait en être le seul élément, d’autant que les poids de naissance ne mettaient pas en exergue de macrosomie. Le recours massif à l’épisiotomie dans notre échantillon pourrait être expliqué par les hypothèses suivantes: 1) pratique des épisiotomies par systématisme, en dépit des recommandations restrictives de 2005 [26]; 2) recours exclusif aux forceps et non à la ventouse obstétricale [27]; 3) hypertonie musculaire périnéale entravant le dégagement du fœtus.

Notre taux d’épisiotomie a pu être amplifié plutôt par une mauvaise application des recommandations que par une hypertonie périnéale due au vaginisme. De manière globale, la diminution de la fréquence des épisiotomies poursuit sa lente décroissance suite à un consensus international sur l’absence de bénéfices d’une épisiotomie systématique, tant en prévention des troubles sphinctériens du postpartum qu’en réponse des professionnels aux demandes des femmes. Cependant, bon nombre des indications des épisiotomies s’avéraient nécessaires du fait d’un vaginisme avéré (4 cas d’hypertonies périnéales et un état de panique conduisant à une extraction sous anesthésie générale). Ainsi, le grand nombre de lésions spontanées constatées peut laisser penser que si les épisiotomies n’avaient pas été pratiquées, il n’est pas certain que nous aurions constaté plus de périnées intacts, contrairement à ce qu’a mis en évidence la littérature pour la population générale [28]. Deux autres études viennent corroborer ce constat [17, 22]. En effet, l’ampliation du périnée nécessite, entre autres, un bon relâchement musculaire, une bonne élasticité des tissus et une absence de peur, trois éléments qui font cruellement défaut chez les femmes atteintes de vaginisme [17, 29].

### En pratique

L’accompagnement d’une femme atteinte de vaginisme comporte des particularités relationnelles. D’abord l’exigence d’une constance et d’une grande patience de la part des professionnels, afin d’éviter les comportements d’évitement et préserver un climat de confiance. Cette relation passe par l’attention que porte ledit professionnel à veiller à ce que la patiente n’éprouve pas le sentiment d’une quelconque perte de contrôle [29]. Force est de constater qu’il s’agit d’un domaine encore méconnu et qu’il n’y a, à l’heure actuelle aucun véritable consensus d’experts. Les éléments recueillis à ce jour incitent à une orientation précoce des femmes atteintes de vaginisme vers une prise en charge sexothérapique qui permet d’agir sur les cognitions, le mécanisme de peur-évitement ainsi que l’hypertonie périnéale conditionnée. Notons que la levée de cette dernière n’est pas suffisante à la réduction des risques d’extractions instrumentales et de morbidité périnéale.

Bon nombre de patientes vaginiques, qui n’évitent que la situation de pénétration, s’accommodent d’une « sexualité externe » qui semble les satisfaire. Dans ce cas de figure, notre description de recherche permanente d’évitement de situation d’intimité, comme c’est le cas des patientes souffrant de désir sexuel hypo-actif, pourrait trouver une certaine limite. Il paraît légitime de donner à ces femmes toutes les chances d’accoucher par voie basse, d’une part pour limiter le surcroit de morbi-mortalité liée à la césarienne [30-32], d’autre part pour ne pas cautionner le comportement d’évitement, qui fait le lit et consolide le vaginisme. Au-delà, l’accouchement par voie basse peut renforcer le sentiment de réussite, et donc le succès d’une sexothérapie en cours [18]. L’exploration du versant proprement psychologique au travers de tests psychométriques (peur, anxiété, dégoût, désir d’enfant, tocophobie) demeure sans doute un domaine à explorer.

### Points forts et limites de l’étude

Cette étude s’est intéressée à un sujet peu traité mais qui a de réelles implications cliniques. Un de ses points forts est qu’il est l’un des rares travaux à s’intéresser aux mécanismes ainsi qu’au déroulement du travail. Une de ses limites tient à son échantillonnage modeste qu’il faut considérer au travers du caractère peu répandu du vaginisme dans la population [33]. Les patientes présentant un vaginisme primaire n’ont été retrouvées que dans 2 études [18, 22], la plupart des travaux intégrant le vaginisme secondaire ou la vulvodynie dont les mécanismes physiopathologiques diffèrent [17, 20, 34]. La méthodologie utilisée n’a sans doute pas autorisé un recrutement exhaustif (inclusion de patientes basée uniquement sur le volontariat des professionnels de santé sollicités); cependant elle a permis d’obtenir une cohorte homogène, apportant une certaine pertinence aux résultats et mettant en lumière plusieurs pistes de réflexion.

Un autre écueil pourrait résider dans la concision du questionnaire qui ne s’intéressait pas expressément aux pathologies obstétricales ni à l’adaptation extra-utérine des nouveau-nés. Le choix délibéré d’un questionnaire court comprenant des questions à compléments multiples permettait de ne pas décourager les professionnels auxquels il était soumis. La place octroyée aux commentaires permettait d’apporter des commentaires et a d’ailleurs été largement utilisée. Enfin, le score de cotation du tonus musculaire périnéal pouvait sembler quelque peu limité pour définir le vaginisme; cependant il a permis de standardiser les mesures du tonus périnéal et le comportement de peur-évitement une fois le diagnostic établi. Cela a par ailleurs permis une utilisation aisée de la part de praticiens qui pouvaient ne pas être familiarisés avec cette entité particulière qu’est le vaginisme.

## Conclusion

Dysfonction importante, le vaginisme est un problème tant individuel que du couple dont il altère la relation sexuelle [35]. Au-delà, il influence péjorativement le pronostic obstétrical. Nos résultats vont dans le sens de ceux rapportés par d’autres auteurs quant au risque accru de césariennes et d’extractions instrumentales pour les femmes atteintes de vaginisme. Ils mettent en outre en lumière le grand nombre de grossesses prolongées, dont on sait qu’elles sont pourvoyeuses de morbidité et de mortalité. L’analyse qualitative des résultats semble désigner le mécanisme de peur-évitement et de l’anxiété, comme éléments constitutionnels essentiels du vaginisme, dans la survenue des dystocies et des lésions périnéales constatées. Un accompagnement sexologique précoce, même s’il ne permet pas à lui seul d’annihiler les risques et améliorer le pronostic obstétrical, pourrait aider ces femmes. La place que l’on pourrait accorder à ce type de prise en charge reste toutefois encore à évaluer au travers d’études prospectives et contrôlées, alliant de plus larges de cohortes de patientes (incluant des tests psychométriques) et explorant le versant néonatal.

### Etat des connaissances actuelles sur le sujet

- Le vaginisme est une pathologie mal connue;
- Peu d’études se sont intéressées au pronostic obstétrical des femmes souffrant de vaginisme.

### Contribution de notre étude à la connaissance

- Le vaginisme influence défavorablement le pronostic obstétrical des femmes vaginiques;
- Cette étude est l’une des rares à avoir enrôlé des patientes enceintes primipares;
- Un prise en charge au cours de la grossesse, notamment psycho-sexologique, pourrait contribuer à améliorer les résultats obstétricaux (taux d’épisiotomie, d’extraction instrumentale et de césariennes en particulier).

## Conflits d’intérêts

Les auteurs ne déclarent aucun conflits d’intérêts.
